# Investigation of factors that cause insulin precipitation and/or amyloid formation in insulin formulations

**DOI:** 10.1186/s40780-019-0151-5

**Published:** 2019-10-30

**Authors:** Yui Ohno, Tomohiro Seki, Yu Kojima, Ryotaro Miki, Yuya Egawa, Osamu Hosoya, Keizo Kasono, Toshinobu Seki

**Affiliations:** 1 Faculty of Pharmacy and Pharmaceutical Sciences, Josai University, 1-1 Keyakidai, Sakado, Saitama 350-0295 Japan; 2 Department of Pharmacy, Japanese Red Cross Medical Center, 4-1-22 Hiroo, Shibuya, Tokyo 150-8935 Japan

**Keywords:** Insulin, Insulin analog, Insulin pump, Multiple daily subcutaneous injections, Continuous subcutaneous insulin infusion, Intravenous solution, Precipitate, Amyloid, Insulin-derived amyloidosis, Insulin ball

## Abstract

**Background:**

Multiple daily subcutaneous injections (MDSIs) are mainly used for formulating an insulin therapy for diabetic patients; however, they also cause insulin-derived amyloidosis (IDA) and lead to poor glycemic control. In addition, for the continuous subcutaneous insulin infusion system (CSII), precipitation frequently causes catheter occlusion and, if the precipitate in the formulations is amyloid, the injection of the insoluble amyloid into the subcutaneous tissue leads to IDA. The aim of this study was to conduct in vitro experiments and present a situation where insulin formulations cause precipitation and amyloid formation.

**Methods:**

Humulin®R and NovoRapid® were used as model formulations for MDSIs and CSII, respectively. The generation of the precipitation was evaluated by measuring turbidity, and amyloid formation was evaluated by using Thioflavin T. Humulin®R was mixed with saline buffer solutions and glucose solutions to evaluate the effect of dilution. In addition, we created an experimental system to consider the effect of the time course of condition changes, and investigated the effects of insulin concentration, *m*-cresol existence, and pH change on the generation of the precipitate and amyloid in the formulation.

**Results:**

In both the original and diluted formulations, physical stimulation resulted in the formation of a precipitate, which in most cases was an amyloid. The amyloid was likely to be formed at a near neutral pH. On the contrary, although a precipitate formed when the pH was decreased to near the isoelectric point, this precipitate was not an amyloid. Further decreases in pH resulted in the formation of amyloids, suggesting that both the positive and negative charged states of insulin tended to form amyloids. The formulation additive *m*-cresol suppressed amyloid formation. When additives were removed from the formulation, the amyloid-containing gel was formed in the field of substance exchange.

**Conclusions:**

To consider changes in conditions that may occur for insulin formulations, the relationship between the formation of precipitates and amyloids was demonstrated in vitro by using insulin formulations. From the in vitro study, *m*-cresol was shown to have an inhibitory effect on amyloid formation.

## Background

Insulin formulations are used to treat type 1 diabetic patients through multiple-daily subcutaneous injections (MDSIs) or continuous subcutaneous insulin infusion (CSII). Currently, commonly-used insulin and insulin analog formulations in a clinical site involve regular insulin formulations, rapid-acting insulin formulations and long-acting insulin formulations, all of these are used for MDSIs and only the rapid-acting insulin formulations are used for CSII. Formulations for individual patients have become possible through various combinations of regular insulin and insulin analogs; in addition, physiological glycemic control has been found to be more optimal for treatment than only the regular insulin formulations. A common mode of administration in the regular insulin and the insulin analog formulations treatment is MDSIs. However, repeated subcutaneous injections of the regular insulin and insulin analog formulations at the same site are known to cause insulin-derived amyloidosis (IDA) in clinical practice [1–15]. IDA leads to a subcutaneous mass, which, because of its shape, is called an “insulin ball” [2]. The absorption of human insulin and its analogs is suppressed when regular insulin and insulin analog formulations are injected into an IDA site; this leads to poor glycemic control and, in some cases, requires increasing doses of the formulations [7, 8]. For example, human insulin and insulin analog levels in blood are reduced by 94% in the IDA site compared to normal sites [5]. Moreover, if the patients inject a higher dose of human insulin or its analogs into a normal site, severe hypoglycemia occurs [8]. It was reported that IDA and its subsequent absorption suppression are caused by the formulations of regular insulin and its analogs. Although site rotation is recommended in MDSIs, the number of reports of IDA is increasing year by year in diabetic therapy [13].

Amyloid formation has been reported in many proteins [16], all of which have a common error in the way they folded. Some proteins, usually with the α-helical conformation, are abnormally folded into β-sheets; such proteins are called amyloids when they are bound to form insoluble fibril/filaments [17, 18]. The formation of fibril/filaments is thought to be related to the processes of the formation and growth of the nucleus [17, 18]. Amyloidosis is a general term for diseases in which insoluble amyloids accumulate in tissues or organs [12]. In the case of human insulin and its analogs, it is presumed that misfolding of their monomers leads to the formation of insoluble insulin amyloids [17, 18].

CSII using the rapid-acting insulins also has the problem of poor glycemic control due to catheter occlusion. According to a clinical study by van Bon et al. unexplained hyperglycemia and/or infusion set occlusion occurred in 61.3–68.4% of patients using CSII [19]. This represents a problem in therapeutics, but the cause of catheter occlusion has not been clarified. Kerr et al. said changes to the conformation and/or properties of the rapid-acting insulin molecules puts them at risk of isoelectric precipitation or fibril formation [20]. Changes in pH, exposure to elevated temperatures, agitation, and/or contact with hydrophobic surfaces can all cause the conformational changes in rapid-acting insulin analogs that promote precipitation, chemical degradation, and/or fibrillation [20]. However, no research has studied them in detail, and there is no report distinguishing between the precipitation of rapid-acting insulin analogs and that of amyloid formation (fibril/filament). IDA can occur if the amyloid precipitates and parts of it end up in the subcutaneous tissue. It is important to determine whether the precipitate is isoelectric or the amyloid not only the regular insulins but also the rapid acting insulins.

Furthermore, the regular insulin formulations are often mixed in with the transfusion in clinical practice [21–24]. The regular insulin formulations are mixed with intravenous nutrition to promote glucose consumption and control, and are administered by intravenous infusion before, during, and after surgery for patients with diabetes [21]. Human insulin precipitation and amyloid formation may be promoted by decreasing concentrations of human insulin itself and the additives (Table 1) in the insulin formulations, interaction with the transfusion ingredients, and change of pH by mixing the regular insulin formulations into the intravenous solution. Therefore, it is clinically important to investigate human insulin precipitation and amyloid formation by mixing the insulin with transfusions.

**Table 1:** Composition and behavior in Humulin®R and NovoRapid®

Formulation	Main medication	pH	Additives (in 100 U/ mL)		
Humulin®R	Human insulin (5807.57)	7.0–7.8	zinc	0.015 mg	(65.38)
			*m*-cresol	2.5 mg	(108.14)
			glycerin	16 mg	(92.09)
			pH regulator	–	–
NovoRapid®	Insulin aspart (5825.54)	7.2–7.6	zinc	0.0196 mg	(65.38)
			*m*-cresol	1.72 mg	(108.14)
			phenol	1.50 mg	(94.11)
			glycerin	16 mg	(92.09)
			disodium hydrogen phosphate dihydrate	1.25 mg	(177.99)
			sodium chloride	0.58 mg	(58.44)
			hydrochloric acid	–	(36.46)
			sodium hydrate	–	(40.00)

The molecular weight or atomic weight is indicated in parentheses

**Fig. 1:**
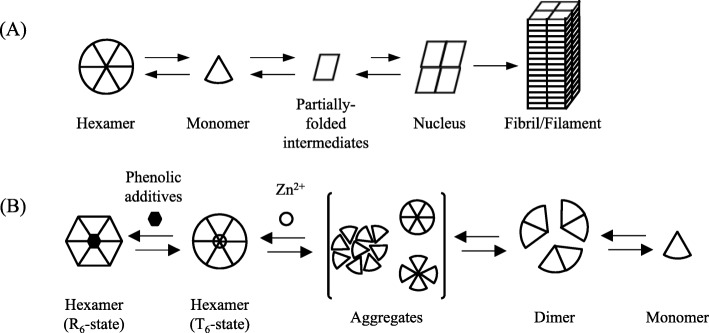
**a** Estimated amyloid formation process of human insulin and its analogs in vitro. **b** Effects of the additives in the insulin formulations on the hexamer formation of insulins. For example, human insulin facilitates the formation of the hexamer in the presence of zinc ions

Amyloid formation in human insulin and its analogs in vitro occurs when the insulin and its analogs form fibrils via partial unfolding of the monomers [17, 18]. Monomers partially unfold, then reassemble to form nuclei, then the nuclei grow into fibril/filaments (Fig. 1a) [17, 18]. Among the additives of the regular insulin formulations, zinc ion and phenolic additives (phenol and/or *m*-cresol) play an important role. Human insulin and its analogs, excluding insulin glulisine, stabilize the hexamers of human insulin and its analogs as an R_6_-state from T_6_-state hexamers (Fig. 1b) [25, 26]. The hexamer of insulin, which contains two zinc ions, takes a tensed form (T_6_-state) that exposes both zinc ions. By adding phenolic additives, the zinc ions are closed to be R_6_-state. Therefore, these additives may also play an important role in preventing amyloid formation of human insulin and its analogs. However, many of the studies on amyloid formation in human insulin and its analogs are studied after excluding the additives from the formulations [27]. From the viewpoint of molecular mechanisms, the additives are not necessary to clarify amyloid formation in human insulin and its analogs themselves. The presence of additives makes it more complicated. It is better to remove additives to know the nature of its folding human insulin and its analogs themselves, however we think that precipitation and amyloid formation of human insulin and its analogs are also important considering effects of the additives, because the additives may have prevent amyloid formation in human insulin and its analogs in the injection site of the patients. In addition, human insulin and its analogs are used in combination with the formulations or mixed with transfusion solutions in clinical practice.

Thus, the aim of this study is to determine in what kinds of clinical situations regular insulin formulations cause precipitation of human insulin; we also determined whether the precipitate is isoelectric or an amyloid. The generation of the precipitate was evaluated by turbidity, and the nature of the precipitate (whether it was an amyloid) was evaluated using thioflavin T (ThT). NovoRapid® was also used as a rapid-acting insulin in some of the experiments. The important thing was to use the formulation to consider *m*-cresol as a phenolic additive. In addition, in order to understand the factors and situations that occur at the clinical site, we created an experimental system that considers the effect of the time course of conditions in the formulations. Specifically, we showed the effects of *m*-cresol on the amyloid formation. The effect of pH change was also discussed.

## Methods

### Simple mixing experiments in a vial

#### The effect of mixing speed

Five mL of Humulin®R (Eli Lilly Japan, Hyogo, Japan) as a regular insulin formulation and NovoRapid® (Novo Nordisk Pharma, Tokyo, Japan) as a rapid-acting insulin analog formulation were shaken (30 rpm) or stirred (850 rpm) in a 6 mL vial at 37 °C to determine effects of physical stimulus on the generation of precipitate in the insulins.

#### The effect of mixing of saline, phosphate-buffered saline, and glucose solutions

One mL of Humulin®R was diluted 5-fold with saline and phosphate**-**buffered saline (PBS, pH 7.4) and stirred (850 rpm) in a 6 mL vial at 37 °C. As the formulation is diluted, the concentration of not only the protein itself but also the *m*-cresol in the formulations decreased. Otsuka normal saline (Otsuka, Tokushima, Japan) was used as the saline.

Five mL of Humulin®R was diluted 5-fold with Otsuka glucose injection (5%, Otsuka) at different pH conditions (original and pH 3.5), and the resulting solutions were stirred (850 rpm) in a 6 mL vial at 37 °C. Hydrochloric acid was added to the Otsuka glucose injection (5%) to adjust it to pH 3.5.

In the vial experiments, the turbidity and ThT response of the sample solutions in the vial were measured over time.

### Experiments using side-by-side diffusion cells

**Fig. 2:**
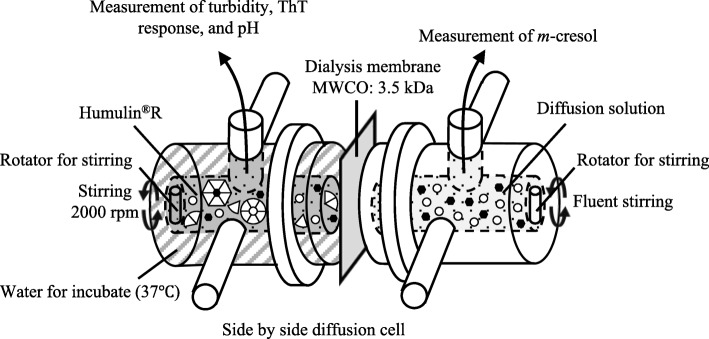
An experimental system in which the composition and pH of the formulation changes over time as the components permeate via the membrane

This experimental system was designed to determine examine the factors related to the generation of precipitate from insulins in the vial experiments. Using the side-by-side diffusion cells, the composition and pH of the experimental solution changes over time as the components diffused via a dialysis membrane and moved into the other cell (Fig. 2). The dialysis membrane (Spectra/Por® Dialysis Membrane, molecular weight cut off (MWCO): 3.5 kDa, SPECTRUM LABORATORIES, California, USA) was pinched between the two half cells. Humulin®R (3.3 mL) was placed in the donor cell and diffusion solutions (volume was same height as the formulation cell) were placed on the other side (Table 2). By choosing the solution in the cell on the other side, the factors that form the precipitate and amyloid could be clarified.

**Table 2:** Experiments using side-by-side diffusion cells

♯	Formulation on the donor side	Solution on the other side	Change in the formulation applied	n
I	Humulin®R	Otsuka normal saline	*m*-Cresol concentration pH	6
II	Humulin®R	PBS (pH 7.4)	*m*-Cresol concentration	5
III	Humulin®R	Otsuka normal saline + *m*-cresol (2.5 mg/mL)	pH	6
IV	Humulin®R	PBS (pH 5.5)	pH, can induce isoelectric precipitation *m*-Cresol concentration	3
V	Humulin®R	PBS (pH 6.5)	pH, cannot induce isoelectric precipitation *m*-Cresol concentration	5

In the diffusion cell experiments, the formulations in the donor side cell were kept at 37 °C and the turbidity, ThT response and pH of the formulations were measured over time. The concentration of *m*-cresol in the other cell was measured to determine what remained in the formulations.

### Preparation of PBS (pH 7.4, 6.5, and 5.5)

The pH was adjusted by adding disodium phosphate (10 mM, FUJIFILM Wako Pure Chemical Corporation, Osaka, Japan) containing sodium chloride (137 mM, FUJIFILM Wako Pure Chemical Corporation) and potassium chloride (2.68 mM, KANTO CHEMICAL, Tokyo, Japan) to potassium dihydrogen phosphate (2.0 mM) containing sodium chloride (137 mM, FUJIFILM Wako Pure Chemical Corporation) and potassium chloride (2.68 mM, FUJIFILM Wako Pure Chemical Corporation).

### Preparation of *m*-cresol-containing saline

An *m*-cresol solution with the same concentration as in Humulin®R was prepared to eliminate the effect of *m*-cresol decrease as another side solution. *m*-Cresol (Sigma-Aldrich Japan, Tokyo, Japan) (250 mg) was added to the Otsuka normal saline (100 mL).

### Measuring turbidity to evaluate the degree of precipitation

Turbidity (λ = 600 nm, 37 °C) was measured by using an ultraviolet visible light spectrophotometer (HITACHI, Tokyo, Japan, U-3000). The sample cuvette was fluent agitated. An elevation in turbidity meant precipitation of solids.

### ThT fluorescence measurement to determine if the precipitate is the simple precipitate or the amyloid

ThT (FUJIFILM Wako Pure Chemical Corporation) is an amyloid-detection reagent that fluoresces in the presence of amyloid. Three mL of the ThT solution (5.0 μM, glycine buffer (50 mM, pH 9.5)) was placed in a cuvette, and a sample in the vial and side-by-side experiments (15 μL) was added, and the fluorescence was measured (λ_ex_ = 444 nm, λ_em_ = 485 nm, room temperature) using a spectrophotofluorometer (SHIMADZU, Kyoto, Japan, RF-5300pc) [28].

### Determination of *m*-cresol in the other side cell

*m*-Cresol in the other side cell in the diffusion cell experiment was measured using High-performance liquid chromatography (HPLC, photo diode array detector: MD-4015, column oven: CO-4061, autosampler: AS-4150, pump: PU-4180) (JASCO, Tokyo, Japan) on C18 column (Mightysil, RP-18 GP 150–4.6 mm, 5 μm). The mobile phase was water containing 25% acetonitrile, the flow rate was 1.2 mL/min, and the column oven was set to 60 °C. The *m*-cresol remaining in the formulations was calculated from the measurement of the amount that permeated the membrane.

### Criteria for precipitate and amyloid formation

When the absorbance at 600 nm was over 0.1, we determined the precipitate generated in the solution; when the fluorescence intensity was over 5, we estimated that the precipitate was an amyloid. If the precipitate formed at near pH 6, we determined that it was isoelectric.

### Statistical analysis

All data are indicated as the mean value ± standard deviation (S.D.). All statistical analyses were computed by using BellCurve for Excel (SSRI, Tokyo, Japan) and statistical significance was evaluated by the application of Student’s *t*-tests for one and two samples, and Tukey’s method for three samples. Differences were considered significant for values of *P* < 0.05.

## Results

### The effect of mixing speed during simple mixing in vial experiments

**Fig. 3:**
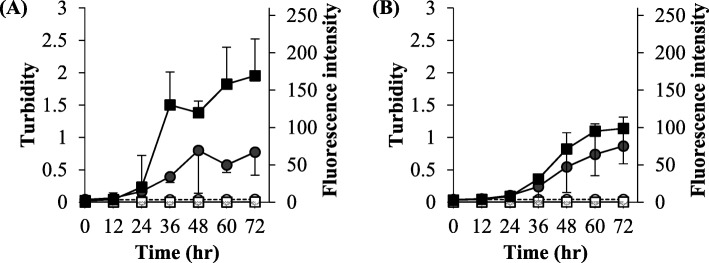
The effect of physical stimulus in the experiments of simple mixing in the vials. For NovoRapid® (**a**) and Humulin®R (**b**), turbidity at 30 rpm (〇) and 850 rpm (●), and fluorescence intensity at 30 rpm (□) and 850 rpm (■) were evaluated. Each value is expressed as the mean ± S.D. (*n* = 3–5)

The turbidity and ThT response of Humulin®R and NovoRapid® did not increase upon weak physical stimulation (30 rpm); however, they increased for both formulations upon strong physical stimulation (850 rpm). At 850 rpm, the elevation of the turbidity and ThT response were simultaneous, suggesting that the precipitate generated was an amyloid. The degree of elevation in turbidity and ThT response were similar for NovoRapid® and Humulin®R (Fig. 3a, b).

### The effect of dilution with saline and PBS (pH 7.4) during simple mixing in vial experiments

**Fig. 4:**
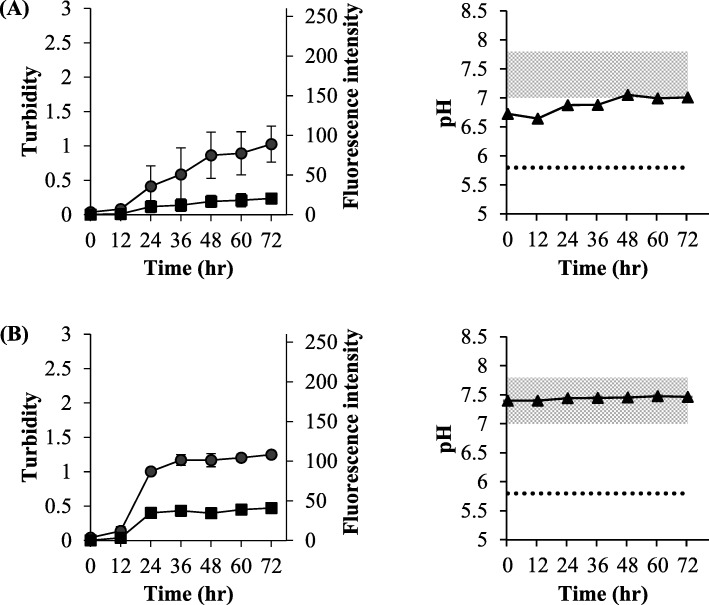
The effect of dilution of Humulin®R in the experiments of simple mixing in vials. Humulin®R was diluted 5-fold with saline (**a**) and PBS (pH 7.4) (**b**). Turbidity (●), fluorescence intensity (■), and pH (▲) were evaluated. The isoelectric point of human insulin in Humulin®R and the pH range in the original formulation of Humulin®R are expressed as a dotted line and filled area in grey. Each value is expressed as the mean ± S.D. (*n* = 3–5). Significantly lower pH value (*) than the lower standard pH value of Humulin®R (*P* < 0.05)

Humulin®R was diluted with saline as a pH-uncontrolled solution and PBS (pH 7.4) as a pH-controlled solution. As in the case of applying a strong physical stimulus (850 rpm), elevation of the turbidity and the fluorescence response were also observed simultaneously for the formulation diluted by saline and PBS (pH 7.4) (Fig. 4). As the ThT response at 24 h for the sample diluted in PBS was significantly higher than that for the undiluted formulations (*P* < 0.05), the dilution may be involved in amyloid formation. The concentration of insulin itself and/or the concentration of *m*-cresol as an additive may be considered as factors that affect amyloid formation. In this experiment, both the dilution with saline and PBS (pH 7.4) resulted in different turbidity and fluorescence response profiles, including different pH values. Therefore, pH may be a factor in the amyloid formation. In the case of saline, turbidity and fluorescence response values were increasing in a sustained manner. The pH of the Humulin®R diluted with saline was 6.5–7.0, slightly lower than that of Humulin®R itself (pH 7.0–7.8). On the contrary, while diluting with PBS (pH 7.4), turbidity and fluorescence response values were increased until 24 h, after which time they were kept constant (Fig. 4b). The pH of the Humulin®R diluted with PBS was approximately pH 7.4, the same as Humulin®R alone.

### Experiments using side-by-side diffusion cells

**Fig. 5:**
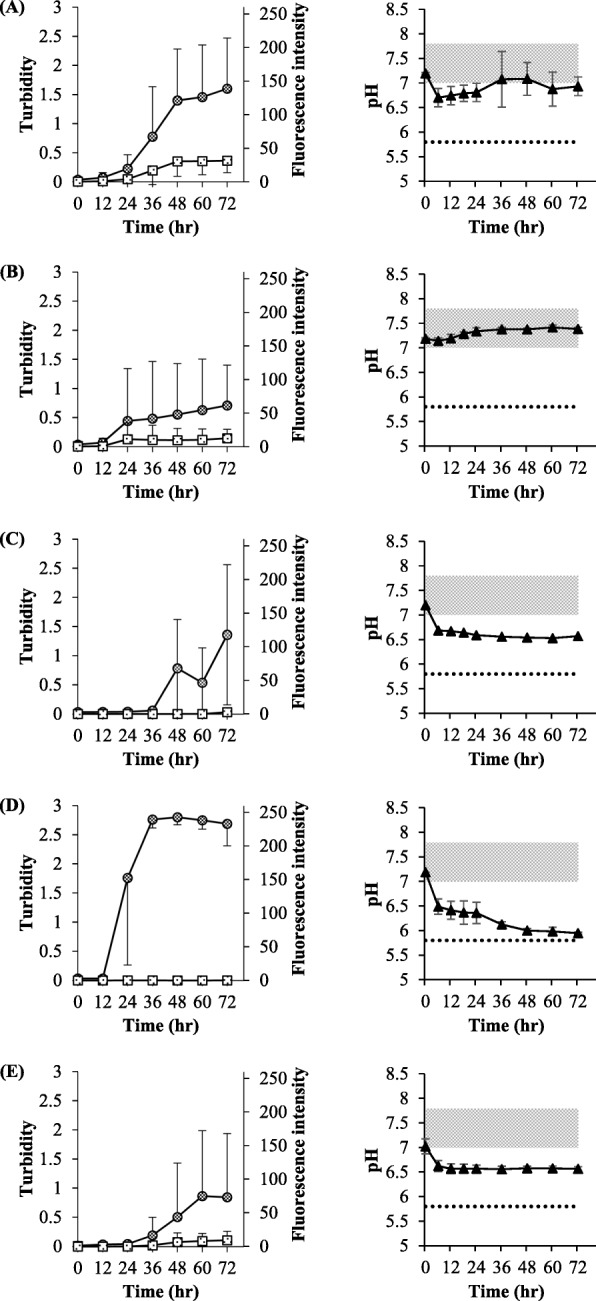
Experiments using side-by-side diffusion cells for Humulin®R. Saline #I (**a**), PBS (pH 7.4) #II (**b**), Saline + m-cresol #III (**c**), PBS (pH 5.5) #IV (**d**), and PBS (pH 6.5) #V (**e**) were applied to the other cell. Turbidity (), fluorescence intensity (), and pH (▲) were evaluated. The isoelectric point of human insulin in Humulin®R and the pH range of the original formulation of Humulin®R are expressed as a dotted line and filled area in grey. Each value is expressed as the mean ± S.D. (*n* = 3–6). Significantly lower pH value (*) than the lower standard pH value of Humulin®R (*P* < 0.05)

**Fig. 6:**
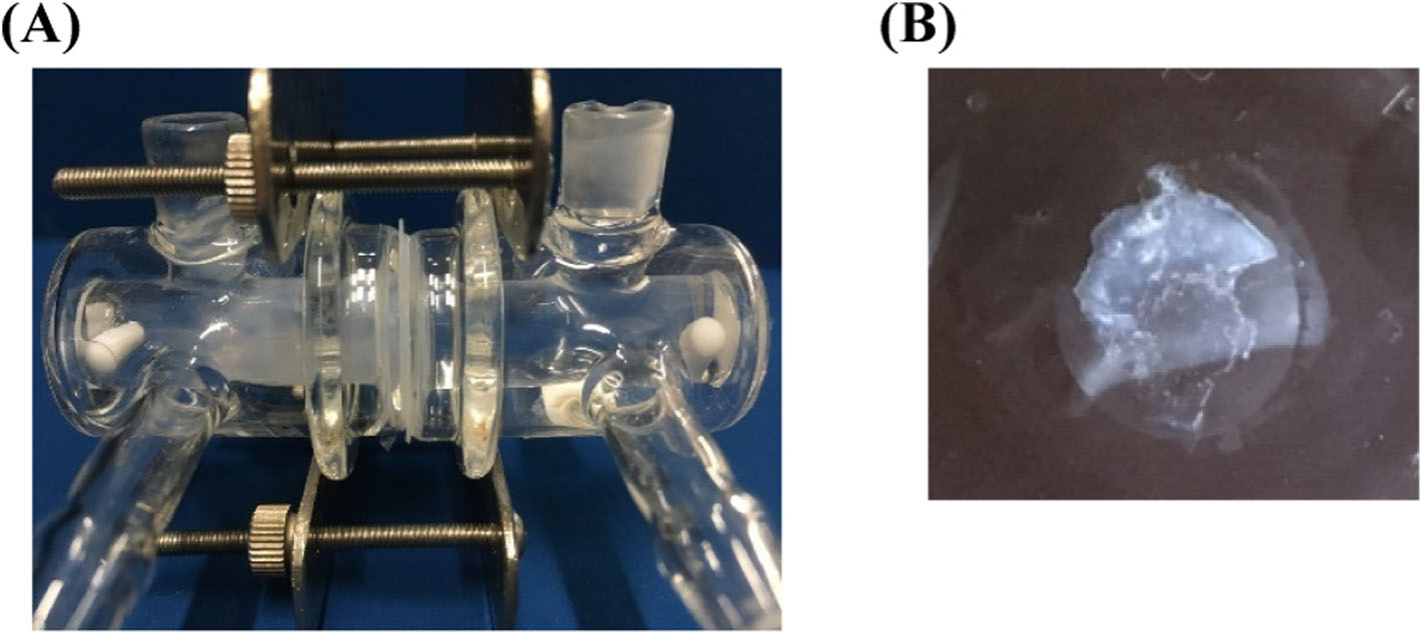
The side view of the cell set at the end of the experiment (**a**) and a photograph of the gel generated (**b**). The gel generated on the membrane donor side when Otsuka normal saline was applied to the other side cell (experiment #I)

In the experiments using side-by-side diffusion cells, if the difference in the components and pH between both the half cells induced migration of the components via the membrane, then the condition in the applied formulation should be changed over time. In the comparison between experiment I (Fig. 5a) and the vial experiment for Humulin®R (Fig. 3b), the increases in the turbidity and the fluorescence intensity were similar; the concentration of insulin itself was not a variable factor for enhanced amyloid formation, because the insulin concentration was kept the same as in the donor cell in experiment I. When the surface of the membrane donor side was observed at the end of the experiments, adhesive gel was present on the surface (Fig. 6). The ThT response of the gel and distant solution from the membrane in the donor cell had fluorescence intensities of 268.6 and 47.0, respectively. The high gel fluorescence means that the gel contains the amyloid fibril/filament. This result suggests that this change in the local condition could induced amyloid formation.

The pH value was kept higher in experiment #II (Fig. 5b by using PBS. The fluorescence intensity increased as the turbidity increased, suggesting that the amyloid was generated similar at pH range 6.5–7.5. In the case of the experiments #I (Fig. 5a) and II (Fig. 5b), the increases in turbidity and fluorescence intensity were similar, suggesting that the decrease in *m*-cresol, and not the pH change, may be the factor controlling amyloid formation.

**Fig. 7:**
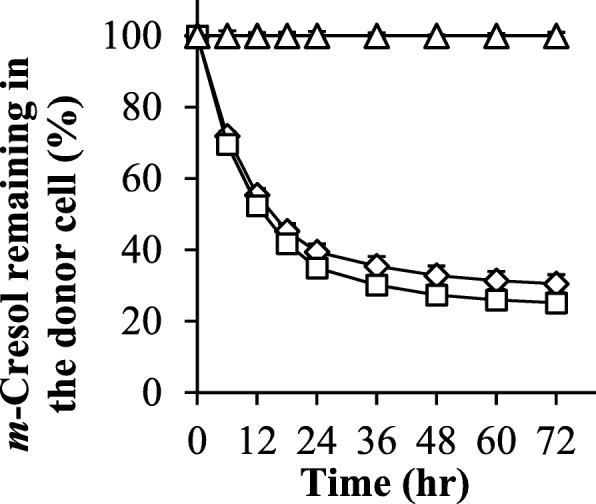
Calculated m-cresol remaining in the donor cell in experiments #I (□), #II (◇) and #III (△). The cumulative amount of m-cresol permeated through the membrane into the other side cell was used for the calculation. Each value is expressed as the mean ± S.D. (*n* = 5–6)

In order to ensure that the contribution of *m*-cresol decreased, the results of experiment #III (Fig. 5c) were compared with those of experiment #I (Fig. 5a). Figure 7 shows the calculated *m*-cresol remaining in the donor cell. In the case of the experiments #I and II, 40% of the *m*-cresol remained after 48 h (Fig. 7). In the case of the experiment #III, turbidity increased after 48 h but the fluorescence intensity did not, suggesting that *m*-cresol had an inhibiting effect on amyloid formation (Fig. 5c). The pH of the donor solution in experiment #III was kept near 6.5.

Experiments #IV (Fig. 5d) and #V (Fig. 5e) were performed to confirm the effects of pH in acidic conditions. pH 5.5 in #IV induced precipitation at the isoelectric point of insulin, but pH 6.5 in #V did not. In experiment #IV, a sharp elevation in turbidity was observed within 36 h, but the fluorescence intensity did not increase (Fig. 5d). The pH decreased to approximately 6, which is near the isoelectric point within 36 h. As the pH decreased to the isoelectric point, the turbidity increased but the fluorescent response did not. This result means that the precipitate at the isoelectric point is different from that at neutral pH and that the precipitate is not the amyloid. On the contrary, in experiment #V, the pH reached 6.5 within 12 h and remained constant thereafter (Fig. 5e). Turbidity and fluorescence intensity simultaneously increased within 36 h. This profile was similar with those of the experiments #I (Fig. 5a) and II (Fig. 5b).

### The effect of dilution with glucose solutions of different pH values during simple mixing in vial experiments

Our experimental results up to this point revealed that pH affects insulin precipitation and amyloid formation. Insulin formulations may be mixed with peripheral parenteral nutrition and total parenteral nutrition. These infusion solutions contain electrolytes, amino acids and glucose, and the pH ranges of those formulation are relatively wide. The wide pH range can affect the generation of insulin precipitation and formation of the amyloid. For example, the Otsuka glucose injection, which is a glucose infusion solution, has a wide pH range (listed as pH 3.5 to 6.5 in the package insert). We prepared an Otsuka glucose injection of pH 3.5 as a model glucose formulation with acidic pH from the untreated pH 4.61–5.08; the untreated and pH-adjusted solutions were used to dilute Humulin®R, and the precipitation and amyloid formation in the mixed solutions were examined.

**Fig. 8:**
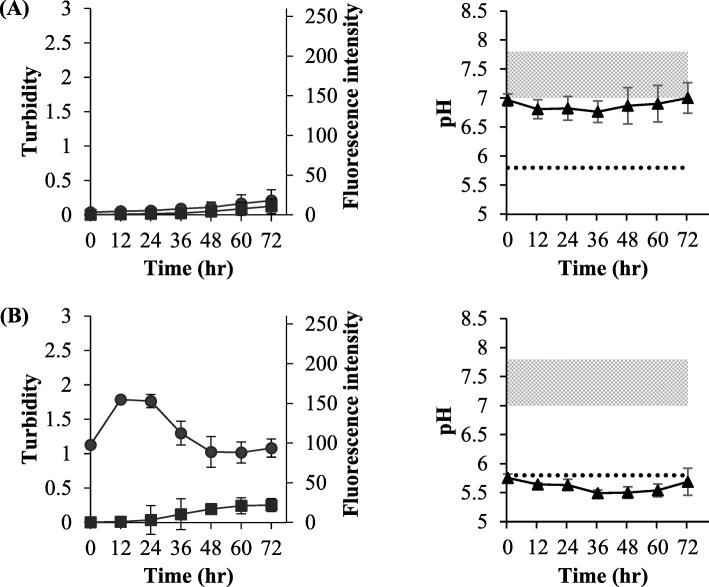
The effect of mixing of Otsuka glucose injection with Humulin®R in the experiments of simple mixing in the vials. Humulin®R was diluted 5-fold with Otsuka glucose injection (5%) (**a**) and Otsuka glucose injection (5%, pH 3.5) (**b**). Turbidity (●), fluorescence intensity (■), and pH (▲) were evaluated. The isoelectric point of human insulin in Humulin®R and the pH range of the original formulation of Humulin®R are expressed as a dotted line and filled area in grey. Each value is expressed as the mean ± S.D. (*n* = 4–10)

For the Otsuka glucose injection at untreated pH (Fig. 8a), the turbidity and ThT response increased simultaneously, suggesting that the precipitate generated was the amyloid. This result was similar to Fig. 4a.

For the Otsuka glucose injection adjusted to pH 3.5 (Fig. 8b), precipitation occurred immediately, but no ThT response was observed. Since the pH at this time was near the isoelectric point, it could be an isoelectric point precipitation. This observation was similar to that shown in Fig. 5d, suggesting that the isoelectric point precipitation did not form the amyloid. The profiles shown in Fig. 8b from 36 to 48 h were different from those shown in Fig. 5d. From 24 h after mixing, the pH further decreased to lower than that of the isoelectric point and the turbidity also decreased, but the ThT response increased (36 to 48 h). This phenomenon occurred because the dissolved insulin in the more acidic conditions formed the amyloid. The promotion of amyloid formation by insulin in acidic conditions has been reported [17].

## Discussion

The purpose of this study was to investigate the factors that cause formulations of insulin and its analogs to precipitate and/or form amyloids, and to examine the clinical situations in which this precipitate and/or amyloid is likely to form. Humulin®R and NovoRapid® were used for the examination. Although many previous studies on amyloid formation used insulin alone and no additives, we considered it important to obtain clinically focused results that take into account additives using the formulation itself, because the additives in the insulin formulations are related to the stability of those insulin formulations during clinical use. Whether the precipitate is an amyloid was evaluated by measuring turbidity and using ThT.

With regard to stimulating applied Humulin® R and NovoRapid®, shaking at 30 rpm did not generate the precipitation, but rapidly mixing at 850 rpm promoted generation of the precipitation, which was regarded as an amyloid (Fig. 3). Although the conditions of 30 rpm and 850 rpm set in this research cannot be directly linked to real conditions, stimulation appears to influence amyloid formation. Since physical stimulation has the potential to be applied in various situations in the daily use of the formulations, Humulin®R and NovoRapid® may change to form the amyloid precipitate through such physical stimulation. For example, physical stimulation such as vibration or shaking occurs during transportation of the formulation, and strenuous driving of an insulin pump may give physical stimulation. Therefore, the formulation should be handled cautiously. Since Humulin®R and NovoRapid® showed similar profiles for amyloid formation, we decided to conduct the subsequent experiments with Humulin®R.

As shown in Fig. 1, the equilibrium state and concentration of insulin affect its hexamer formation, and *m*-cresol acts as an additive to stabilize the hexamer. Humulin®R diluted with saline and PBS (pH 7.4), under the strong physical stimulation in the vial (Fig. 4), accelerated amyloid formation compared to the undiluted solution (Fig. 3b). By diluting the Humulin®R, the formulation lowered not only its protein concentration, but also the concentration of the additive; we performed the experiments using side-by-side diffusion cells to understand these phenomena for the experiments of simple mixing in the vial. Since the formation of the amyloid in the insulin formulations was suppressed by the existence of *m*-cresol as the comparison between experiments #I (Fig. 5a) and #III (Fig. 5c), it is likely that the equilibrium shift to generate the monomer shown in Fig. 1b could be the important step to form the amyloid. The stable hexamer is in the R6 state when *m*-cresol is present, and zinc ion loss from the hexamer could be suppressed by protection with the *m*-cresol. The disappearance of *m*-cresol could be causes the release of zinc ion, after which the equilibrium shifts in the direction of dissociation into monomers.

The effect of pH on the amyloid formation was apparent from the results of experiments #II (Fig. 5b), #IV (Fig. 5d), and #V (Fig. 5e) and Fig. 8. Since experiments #II and #V, in which the pH is kept near neutral, showed similar results, the amyloid is likely to form at a pH that is higher than that of the isoelectric point. On the contrary, the results in experiment #IV, which decreased to near the isoelectric point, and Fig. 8b, in which the pH was equal to that of the isoelectric point immediately after dilution, suggested that the precipitate the formed at the isoelectric point was not an amyloid. At 24 to 72 h in Fig. 8b, lower pH than the isoelectric point, decreasing turbidity, and increasing ThT response were observed. Since it has been reported that amyloid formation is promoted at low pH, the profiles in Fig. 8b might mean that the precipitate that dissolved at the acidic condition changed to form the amyloid [17]. It should be noted that the same phenomenon may occur when mixing solutions into transfusions.

In this study, the formation of precipitate was evaluated by turbidity measurement, ThT fluorescence measurement was used to determine whether the precipitate was amyloid. We determined that the precipitate at the isoelectric point was not an amyloid. However, since the turbidity does not indicate the amount of insoluble proteins accurately, we have to notice that the results in this study were semi-quantitative. In addition, although amyloids of proteins have been reported to form different structures depending on pH, ThT does not give information on their structure and, in this study, the structure of the amyloids observed are not clear. Amyloid structure should be investigated in the future.

The experimental system using the side-by-side diffusion cells we made has the following advantages. The effects of additives and solvents on insulin denaturation can be investigated by applying various solutions to the cell that is separated from the donor cell that contains the formulation, and the conditions of the formulation’s gradual change over time. This dynamic process can be controlled and the kinetics can be evaluated easily using different membranes. In addition, this experimental system using side-by-side diffusion cells can be used as a model system simulating the in vivo conditions of subcutaneous spaces. We observed the gel formed at the surface of the membrane on the donor side (Fig. 6), and the gel was found to contain amyloids. The gel was formed near the membrane where diffusion and migration of the components occurred. Such a gel can develop in an injected tissue when components of the injected the formulations move out via capillary vessels. Since subcutaneously injected insulins have the highest molecular weight (MW) in the formulations, the insulins remain in the subcutaneous tissue because of low diffusion coefficient (*D*), whereas the other components diffuse and move out. When *D* values were calculated based on the corresponding MW, the *D* value of human insulin (1.1 × 10^− 6^ cm/s) was 10% of that of *m*-cresol (1.1 × 10^− 5^ cm/s) [29, 30]. Under such conditions, insulins can form the gel and change to form amyloids. While we obtained important basic results in vitro, it is also useful to understand the formation of amyloids of insulins in vivo.

## Conclusions

This is the first study to evaluate insulin precipitation or amyloid formation in insulin formulations considering the effects of the additives in the formulations. This experimental approach is valuable for pharmacists preparing insulin formulations and advising patients regarding these medications.

In both the normal and diluted formulations, physical stimulation resulted in enhanced insulin precipitation. *m*-Cresol as the additive in a formulation suppressed the formation of amyloid. Amyloid formation occurs at a different pH from the isoelectric point. Precipitation also occurs at the isoelectric point, but it was not due to amyloid formation.

Although we used ThT to determine whether the precipitate was an amyloid or not, since the formation of a precipitate itself is a problem in clinical practice, all results of this study provide necessary information for pharmacists advising patients regarding insulin medications.

## Data Availability

The datasets supporting the conclusions of this article are included within the article.

## Abbreviations

CSII: Continuous subcutaneous insulin infusion
*D*: Diffusion coefficient
IDA: Insulin-derived amyloidosis
MDSIs: Multiple daily subcutaneous injections
MW: Molecular weight
MWCO: Molecular weight cut off
PBS: Phosphate buffer saline
ThT: Thioflavin T

## References

[1] Störkel S, Schneider HM, Müntefering H, Kashiwagi S (1983). Iatrogenic, insulin-dependent, local amyloidosis. Lab Investig.

[2] Nagase T, Katsura Y, Iwaki Y, Nemoto K, Sekine H, Miwa K (2009). The insulin ball. Lancet..

[3] Yumlu S, Barany R, Eriksson M, Röcken C (2009). Localized insulin-derived amyloidosis in patients with diabetes mellitus: a case report. Hum Pathol.

[4] Sie MPS, van der Wiel HE, Smedts FMM, de Boer AC (2010). Human recombinant insulin and amyloidosis: an unexpected association. Neth J Med.

[5] Tomoyuki Y, Munehiro H (2012). Insulin absorption inaPatient with local subcutaneous amyloid deposition. J Japan Diabetes Soc..

[6] Okamura S, Hayashino Y, Kore-Eda S, Tsujii S (2013). Localized amyloidosis at the site of repeated insulin injection in a patient with type 2 diabetes. Diabetes Care.

[7] Yabe S, Takahashi H, Gotoda H, Mori T, Ikawa H, Kudo H (2015). A case of type 1 diabetes mellitus with marked poor glycemic control caused by insulin-derived Amyloidosisat the site of repeated insulin injections. J Japan Diabetes Soc..

[8] Nagase T, Iwaya K, Iwaki Y, Kotake F, Uchida R, Oh-I T (2014). Insulin-derived amyloidosis and poor glycemic control: a case series. Am J Med.

[9] Keiko Y, Chiharu T, Kamimura S, Naoko I, Ayako Y, Hiroko I (2015). The importance of subcutaneous mass detection in insulin injection sites and the guidance of site rotation. J Japan Diabetes Soc.

[10] Kazuhisa K, Tomoyuki Y, Tomoko T, Munehiro H (2015). The analysis of histology, imaging findings, and insulin absorption about subcutaneous masses which occurred in insulin injection sites. J Japan Diabetes Soc..

[11] Bernárdez C, Schärer L, Molina-Ruiz AM, Requena L (2015). Nodular amyloidosis at the sites of insulin injections. J Cutan Pathol.

[12] Gupta Y, Singla G, Singla R (2015). Brief communication insulin - derived amyloidosis. Indian J Endocrinol Metab.

[13] Nilsson MR (2016). Insulin amyloid at injection sites of patients with diabetes. Amyloid..

[14] Hagiwara Seiya, Taneda Shinji, Fukumoto Takaya, Hagiwara Kazuya, Kikuchi Minoru, Kimura Tetsunori, Nakayama Hidetaka, Manda Naoki (2017). Localized Subcutaneous Insulin-Derived Amyloidosis Excised after Evaluation Using Ultrasonography in a Patient with Type 2 Diabetes Mellitus. Case Reports in Endocrinology.

[15] Ansari AM, Osmani L, Matsangos AE, Li QK (2017). Current insight in the localized insulin-derived amyloidosis (LIDA): clinico-pathological characteristics and differential diagnosis. Pathol Res Pract.

[16] Sipe JD, Cohen AS (2000). Review: history of the amyloid fibril. J Struct Biol.

[17] Nielsen L, Frokjaer S, Brange J, Uversky VN, Fink AL (2001). Probing the mechanism of insulin fibril formation with insulin mutants. Biochemistry..

[18] Yang Y, Petkova A, Huang K, Xu B, Hua QX, Ye IJ (2010). An Achilles’ heel in an amyloidogenic protein and its repair: insulin fibrillation and therapeutic design. J Biol Chem.

[19] Randomized DA, Trial C, Charpentier G. Insulin Glulisine compared to insulin Aspart and to insulin Lispro administered by continuous subcutaneous insulin infusion in patients with type 1. Dianetes Technology & Therapeutics. 2011;13:607–14. 10.1089/dia.2010.0224 21457066

[20] Kerr D, Wizemann E, Senstius J, Zacho M, Ampudia-Blasco FJ, Affiliations A (2013). Stability and performance of rapid-acting insulin analogs used for continuous subcutaneous insulin infusion: a systematic review. J Diabetes Sci Technol.

[21] Matsubara H, Yago K (2009). Absorption of medicine to in-line filter. ZYOUMYAKUKEITYOUEIYO..

[22] Kohno E, Izumi N, Toyoda T (2012). Effects of ingredients and pH of intravenous solutions on stability of insulin. J Pharm Heal Care Sci.

[23] Kohno E, Izumi N, Yasunaga H, Nakamura N, Matsumoto E, Okuyama E (2012). Insulin stability in peripheral parenteral nutrition solutions. J Pharm Heal Care Sci..

[24] Mizumoto K, Fuke H, Hashimoto A, Kawazoe F, Sakuma T, Yamagata M (2018). The necessity of blood glucose monitoring during total parenteral nutrition of non-daibetic ptients. J Soc Parenter Enter Nutr.

[25] Brange J., Owens D. R., Kang S., Volund A. (1990). Monomeric Insulins and Their Experimental and Clinical Implications. Diabetes Care.

[26] Birnbaum DT, Kilcomns MA, Defelippis MR, Beals JM (1997). Assembly and dissociation of human insulin and LysB28ProB29-insulin hexamers a comparison study. Pharm Res.

[27] Woods R. Jeremy, Alarcón Javier, McVey Elaine, Pettis Ronald J. (2012). Intrinsic Fibrillation of Fast-Acting Insulin Analogs. Journal of Diabetes Science and Technology.

[28] Kitagawa K, Misumi Y, Ueda M, Hayashi Y, Tasaki M, Obayashi K (2015). Inhibition of insulin amyloid fibril formation by cyclodextrins. Amyloid..

[29] Seki T, Mochida J, Okamoto M, Hosoya O, Juni K, Morimoto K (2003). Measurement of diffusion coefficients of parabens and steroids in water and 1-octanol. Chem Pharm Bull.

[30] Hosoya O, Chono S, Saso Y, Juni K, Morimoto K, Seki T (2004). Determination of diffusion coefficients of peptides and prediction of permeability through a porous membrane. J Pharm Pharmacol.

